# Zinc-Based Nanoparticles Reduce the Bacterial Burden and Protect Collagen in a Mouse Cutaneous Wound Model

**DOI:** 10.1021/acsomega.5c06287

**Published:** 2026-01-24

**Authors:** Rafael Bianchini Fulindi, Thulio Wliandon Lemos Barbosa, Vanessa Enriquez, Claudia L. Charles-Niño, Natália Galvão de Freitas, Mariana Picchi Salto, Leila Aparecida Chiavacci, Sebastião Pratavieira, João Pessoa Araújo Junior, Paulo Inácio da Costa, Luis R. Martinez

**Affiliations:** 1 Department of Clinical Analysis, São Paulo State University (UNESP), Araraquara, São Paulo 14800-903, Brazil; 2 Department of Drugs and Medicines, 28108 UNESP, Araraquara, São Paulo 14800-903, Brazil; 3 Department of Oral Biology, 164889 University of Florida College of Dentistry, Gainesville, Florida 32610, United States; 4 São Carlos Physics Department, University of São Paulo, São Carlos, São Paulo 13500-000,Brazil; 5 Biotechnology Institute, São Paulo State University, Botucatu, São Paulo 18618-970,Brazil; 6 Emerging Pathogens Institute, University of Florida, Gainesville, Florida 32610,United States; 7 Center for Immunology and Transplantation, 3463 University of Florida, Gainesville, Florida 32610,United States; 8 McKnight Brain Institute, University of Florida, Gainesville, Florida 32610,United States; 9 Center for Translational Research in Neurodegenerative Disease, University of Florida, Gainesville, Florida 32610,United States

## Abstract

The persistent threat of multidrug-resistant bacteria, particularly within biofilms, continues to undermine conventional antimicrobial therapies. In this study, we explored the potential of zinc oxide (ZnO) and zinc sulfide (ZnS) nanoparticles (NPs) as alternative strategies to target clinically relevant bacteria such as *Staphylococcus aureus*, *Klebsiella oxytoca*, and *Pseudomonas aeruginosa*. Both NPs exhibited effective antibacterial activity against planktonic forms, with ZnO more potent *in vitro*. However, ZnS-NPs were more efficacious in disrupting mature biofilms by compromising their metabolic activity. Scanning electron and confocal microscopy revealed that Zn-NP treatment compromised the structural integrity of the biofilms. ZnS-NPs also triggered a marked downregulation of genes associated with *P. aeruginosa* exopolysaccharide biosynthesis (e.g., *pslA* and *algC*), suggesting specific interference in biofilm formation pathways. Topical treatment of skin wound infection in Balb/c mice led to a significant reduction in bacterial burden. Notably, while Zn-NPs did not promote initial wound healing, they inhibited the degradation of collagen by bacteria and/or helped maintain collagen levels in the skin of the mice. These findings demonstrate that Zn-NPs effectively reduce early bacterial burden in mouse skin wounds while preserving cutaneous tissue collagen integrity, establishing their dual therapeutic potential as both antimicrobial and tissue-protective agents in wound management.

## Graphical abstract

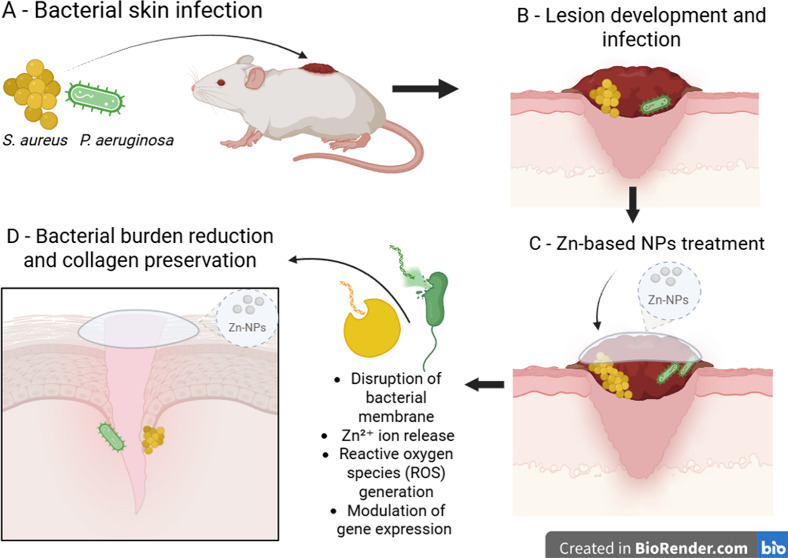

## Introduction

Most bacteria of medical importance can form biofilms, which are frequently difficult to treat. Biofilms provide bacteria with a remarkable ability to survive and colonize the host due to their high resistance to antibiotics and immune response.^1^ Bacterial infections represent one of the leading causes of mortality in children globally, with ∼ 550,000 neonatal deaths worldwide annually, mostly in low- and middle-income countries.^2^ In nations with more robust health systems, the economic impact of bacterial infections is considerable. For example, Healthcare-Associated Infections (HAIs) cost between $28 and $45 billion annually in the United States (U.S.).^3^ Antimicrobial resistance significantly increases this burden, adding ∼ $1,383 to the treatment of a bacterial infection in adults, totaling up to $2.2 billion annually.^4^ Patients in intensive care units (ICU), particularly children, exhibit increased vulnerability to HAIs, with bloodstream infections being the most common and strongly linked to the use of invasive medical devices.^5^ HAIs affect ∼ 50% of patients with indwelling or prosthetic devices used for medical treatment including catheters, cardiac pacemakers, joint replacements, dental prostheses, prosthetic heart valves, and contact lenses.^6^ These medical devices can be easily colonized by microbes because they provide an ideal surface for microbial cell adhesion. Biofilm-forming bacteria are associated with many infections such as endocarditis, osteomyelitis, sinusitis, urinary tract infections, chronic prostatitis, periodontitis, otitis media, and cystic fibrosis.^7^ Due to their antibiotic resistance, *Staphylococcus aureus*, *Klebsiella oxytoca*, and *Pseudomonas aeruginosa*, are listed as high priority bacteria by World Health Organization,^8^ and their ability to form biofilms are a threat to public health.

The Gram-positive bacterium *S. aureus* is a commensal of humans, isolated from 80% of individuals with HAIs.^9^ Given to its close association with the human host, *S. aureus* produces many virulence factors that enhance its ability to cause disease including toxins (e.g., pore-forming, exfoliative, superantigens) and extracellular enzymes (e.g., coagulase, lipase).^10^ Moreover, *S. aureus* can form biofilms in host tissue after adhesion to tissue (e.g., heart valves, bone) or medical implants (e.g., catheters, prosthetic joints, and pacemakers), and multiply producing a protective exopolymeric matrix that encases the bacterial community. This ability allows *S. aureus* to colonize and persist within host tissue, often leading to chronic infections due to immune cell and antibiotic resistance.^11^ In this regard, antibiotic-resistant *S. aureus* infections cost ∼ $2 billion and kills >10,000 U.S. patients per year.^12^

*P. aeruginosa* and *K. oxytoca* are Gram-negative bacteria with high resistance to commonly used antibiotics and their HAIs are challenging to treat resulting in high morbidity and mortality. *P. aeruginosa* causes opportunistic and severe disease in immunosuppressed patients, including those with cystic fibrosis or wounded/burned,^13^ due to its ability to form strong biofilms and express numerous virulence factors. *K. oxytoca* is a commensal frequently found in the nasopharyngeal and intestinal tract of humans and can cause opportunistic disease in patients with compromised immunity.^14^ Outbreaks of multidrug-resistant *K. oxytoca* have occurred in hospitals around the world,^15^ particularly in long-term care facilities and ICUs,^16^ where handwashing stations have been identified as a possible environmental reservoir.^17^ Moreover, *K. oxytoca* produces cytotoxins that can cause postantibiotic diarrhea and colitis^18^ in younger^19^ and older^20^ immunosuppressed individuals. *K. oxytoca* isolates from patients with antibiotic-associated hemorrhagic colitis^21^ and colorectal cancer^22^ showed high adhesin expression and biofilm formation. Hence, novel therapeutic and treatment strategies are urgently needed to combat infections by Gram-negative bacteria, especially those with natural abilities to form biofilms. Most of the bacterial strains used in this study, including clinical isolates, are resistant to commonly used antibiotics (Table 1), highlighting the urgent need for alternative antimicrobial strategies.

**Table 1:** Bacterial Strains Used in These Studies

bacteria	strains
*S. aureus*	ATCC 25923, 553838, 553849^a^, 9^b^, 127^b^, 114–6^b^, 102–9^b^
*K. oxytoca*	ATCC 13182, MIT 10–5249^b^, MIT 09–7231^b^, MIT 10–5250^b^, MIT 10–5246^b^
*P. aeruginosa*	ATCC 27853, MRSN 5519, MX 0560^b^, MRSN 1601^b^, MRSN 11281^b^

a Clinical strains used *in vivo*.

b Methicillin-resistant strains.

We recently demonstrated that zinc (Zn)-based nanoparticles (NPs) reduce *S. aureus*, *K. oxytoca*, and *P. aeruginosa* biofilm formation *in vitro*.^23^ ZnO- and ZnS-NPs inhibit bacterial viability and proliferation via generation of reactive oxygen species (ROS) causing damage to cellular components (e.g., lipids, proteins, and DNA), release of Zn^2+^ ions inducing toxicity through apoptosis,^24^ and/or directly affecting the expression of virulence factors. While these mechanisms are supported by previous literature,^25^ we did not directly measure ROS activity or protein secretion in our study. Nonetheless, the observed reduction in bacterial viability and biofilm formation across multiple strains provides functional evidence of the antibacterial potential of Zn-NPs. In this study, we investigated the efficacy of Zn-NPs in reducing bacterial viability, gene expression, and biofilm formation. Using a validated mouse model of wound infection,^26–28^ we demonstrated that Zn-NPs reduce wounded tissue bacterial load and alter microbial biofilm-associated gene expression. Although Zn-NPs did not stimulate early wound healing, they prevented bacterial collagen degradation and/or preserved collagen in the mouse skin. Our findings suggest the possibility of future use of Zn-based NPs in the treatment of skin infections.

## Results

### Structural Characteristics of the Zn-NPs Generated Using the Sol–Gel Method

We generated ZnO- and ZnS-NPs using the sol–gel methodology described by Spanhel and Anderson^29^ and Manaia et al.,^30^ respectively, with a few modifications. To characterize the structure of ZnS and ZnO-NPs, we utilized X-ray diffraction (XRD) and UV–vis spectroscopy (Figure 1). The XRD of ZnO-NPs revealed the hexagonal crystalline shape with the presence of the corresponding planes 100, 101, 102, 110, 103, and 102 (Figure 1A). The noise observed in the XRD, in addition to the broadened peaks, can be explained by the size of the primary NP, which was calculated using the Scherrer equation for crystal size determination, showing an average size of 5.2 nm. UV–vis spectroscopic analysis of ZnO-NPs showed an absorption of 347 nm (Figure 1B), validating previous reported results.^29^

**Figure 1:**
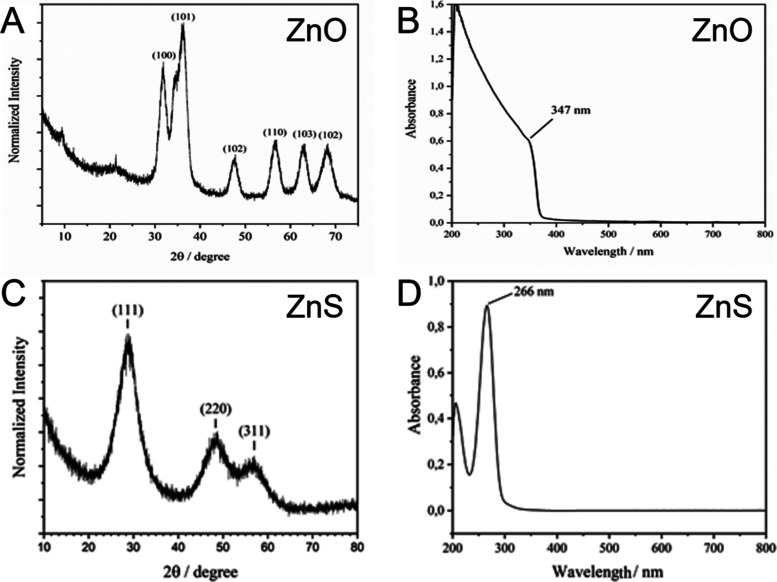
Characterization of Zn-based nanoparticles (NPs) prepared by the sol–gel method. (A) X-ray diffraction (XRD) spectra of ZnO-NPs, revealing a hexagonal crystalline formation with corresponding planes around 100. The broadened peaks indicate a primary NP size of 5.2 nm, as calculated by the Scherrer equation. (B) UV–vis absorption spectra of ZnO-NPs dispersed in ethanol, showing a characteristic shoulder in the region of 350 nm. (C) XRD spectra of ZnS-NPs, showing Bragg peaks at positions 29.04°, 48.32°, and 56.60° 2θ, attributed to the symmetry planes of the cubic ZnS structure. The average crystal size was estimated to be 1.37 nm using the Scherrer equation, indicating a cubic crystalline phase. (D) UV–vis absorption spectra of ZnS-NPs dispersed in ethanol, with an absorption band at 266 nm, characteristic of the formation of ZnS in its cubic crystalline phase.

For ZnS-NPs, the Bragg peaks at positions 29.04°, 48.32°, and 56.60° (2θ) shown in the XRD corresponded to the 111, 220, and 311 symmetry planes of the cubic Zn blende structure of ZnS (Figure 1C), as previously reported.^31^ The average crystallite size was calculated using the Scherrer equation: L = Kλ/(β cosθ); where L is the crystallite size in nm, β is the full width at half-maximum of the peak, in radians, λ is the wavelength of the X-ray beam in nm, and θ is the Bragg angle of the peak analyzed in radians. The crystallite size estimated by the peak at 29.04° 2θ was 1.37 nm. These results showed that the method adopted for synthesizing ZnS-NPs had a cubic crystalline phase, but with a reduced size when compared to other methodologies.^32^ In the absorption spectrum in the UV–vis region (Figure 1D), there is an absorption band at 266 nm that is characteristic of the formation of ZnS in its cubic crystalline phase.^31^ According to the study by Chen et al.,^32^ the spectrum may also show a redshift depending on the crystallite size. Zeta potential measurements were also carried out to confirm the ZnS-NP preparation by dispersing them in water with 0.5% DMSO (data not shown). ZnS-NPs charge was – 2.31 ± 0.35 mV, which is in line with the data found in the literature for ZnS in acidic media.^33^ In aqueous media with a pH between 2 and 4, ZnS has negative zeta potential values close to 0. In addition, the sulfur-rich suspension can form H_2_S in acidic solution.^33^ These results validate that our ZnO^29^ and ZnS^30^ NP preparations were structurally similar to those previously described.

The cytotoxicity of ZnS- and ZnO-NPs was evaluated *in vitro* using MTT and Neutral Red assays in exposed Huh-7.5 human hepatocarcinoma cells (data not shown). ZnS-NPs maintained high cell viability (>90%) across the tested concentrations of 2.5–100 μg/mL, demonstrating minimal cytotoxic effects. Similarly, ZnO-NPs exhibited dose-dependent cytotoxicity, with reduced cell viability observed at concentrations above >10 μg/mL. These results align with published literature reporting the low toxicity of ZnS-NPs and the moderate toxicity of ZnO-NPs in mammalian cells,^34–36^ confirming their favorable safety profile for our studies.

### ZnO- and ZnS-NPs Inhibit Growth of Medically Important Planktonic Bacteria

Given the medical importance of *S. aureus*, *K. oxytoca*, and *P. aeruginosa*, we determined the minimum inhibitory concentration (MIC_50_) of the Zn-NPs required to inhibit the growth of 50% of the bacterial population (Figure 2). We determined the MIC_5__0_ of Zn-NPs across multiple strains of *S. aureus*, *K. oxytoca*, and *P. aeruginosa*, including the standard strains *S. aureus* ATCC 25923, *K. oxytoca* ATCC 13182, and *P. aeruginosa* ATCC 27853 as well as in several clinical antibiotic-resistant strains (Table 1.)

**Figure 2:**
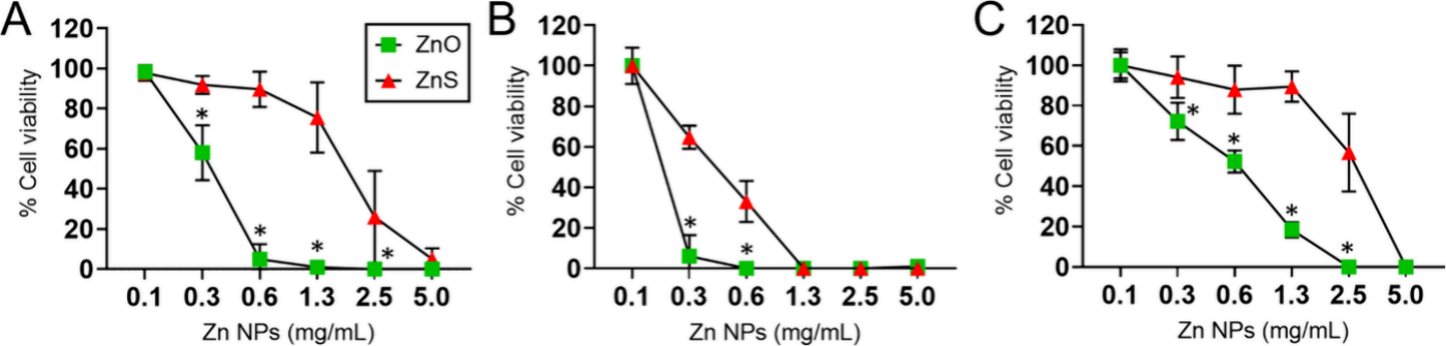
Minimal inhibitory concentration (MIC_5__0_) of ZnO- and ZnS-NPs required to inhibit planktonic bacteria. MIC_5__0_ values were determined for multiple strains of each species (Table 1): (A) *Staphylococcus aureus* (*n* = 7 strains), (B) *Klebsiella oxytoca* (*n* = 5 strains), and (C) *Pseudomonas aeruginosa* (*n* = 5 strains). Symbols indicate the mean ± standard deviation of all strains tested. NPs-treated bacteria were compared to untreated bacteria, and percentage (%) cell viability was calculated as % cell viability = (NPs-treated bacteria/untreated bacteria) × 100. Symbols and error bars denote the means and standard deviations (SDs), respectively. Asterisks indicate *P*-value significance (* *P* < 0.05) calculated using multiple students’ *t*-test analysis.

The MIC_50_ of ZnO- and ZnS-NPs against *S. aureus* were 0.6 and 2.5 mg/mL, respectively (Figure 2A). ZnO-NPs were more effective than ZnS-NPs in inhibiting the growth of *S. aureus* at concentrations ≥ 0.3 mg/mL (*P* < 0.05). Less than 5% of *S. aureus* cells survived after exposure to 0.6 mg/mL ZnO-NPs, and the growth was completely inhibited at concentrations ≥ 1.3 mg/mL. ZnS-NPs at ≥ 5 mg/mL inhibited the growth of all *S. aureus* cells. *K. oxytoca* was the most susceptible bacterium to Zn-NPs treatment (Figure 2B). The MIC_50_ of ZnO- and ZnS-NPs were ≥ 0.3 and 0.6 mg/mL against *K. oxytoca*, respectively. The percentage of *K. oxytoca* viability was dramatically reduced after incubation with 0.3 mg/mL of ZnO-NPs and completely reduced after exposure to 0.6 mg/mL. Exposure of *K. oxytoca* cells to 0.3 and 0.6 mg/mL ZnO-NPs more significantly reduced their viability relative to similar ZnS-NPs concentrations (*P* < 0.05). All *K. oxytoca* cells were inhibited by ZnS-NPs after exposure to 1.3 mg/mL. *P. aeruginosa* was the most resistant of all bacterial species tested (Figure 2C). The MIC_50_ for *P. aeruginosa* after treatment to ZnO- and ZnS-NPs was 1.3 and 5 mg/mL, respectively, with ZnO-NPs being more effective at ≥ 0.3 mg/mL than ZnS-NPs in reducing *P. aeruginosa* viability (*P* < 0.05). The complete growth inhibition of *P. aeruginosa* cells occurred after ZnO- and ZnS-NPs incubations of 2.5 and 5 mg/mL, respectively. Our results indicate differential efficacy between ZnO- vs ZnS-NPs in reducing the viability of medically important bacteria.

### ZnS-NPs Were More Effective than ZnO-NPs in Reducing the Metabolic Activity of Mature Bacterial Biofilms

Microbial biofilms are significantly more resistant to environmental stress and antimicrobial drugs. Therefore, we evaluated and compared the efficacy of Zn-NPs to mature bacterial biofilms using standard and clinical strains (Table 1) and the XTT reduction assay, which is a semiquantitative test that measures cellular metabolic activity of biofilms after the conversion of XTT to formazan salt by the electron transport chain. The biofilms were treated with Zn-NPs at concentrations according to the MIC_5__0_ previously determined for each strain (Figure 2), ensuring that the evaluation considered the intrinsic susceptibility of each bacterial species and isolate. Contrary to what we observed in planktonic bacteria, ZnS-NPs were more efficacious in reducing the metabolic activity of bacterial cells within biofilms compared to ZnO-NPs (Figure 3). We observed that five out of seven *S. aureus* (Figure 3A), all four *K. oxytoca* (Figure 3B), and four out of six *P. aeruginosa* (Figure 3C) strains were more susceptible to ZnS than to ZnO-NPs (*P* < 0.05). Only 2 isolates of *S. aureus* (isolates 2 and 3; Figure 3A) and *P. aeruginosa* (isolates 1 and 6; Figure 3C) exhibited comparable reductions in metabolic activity after incubation with both types of Zn-NPs.

**Figure 3:**
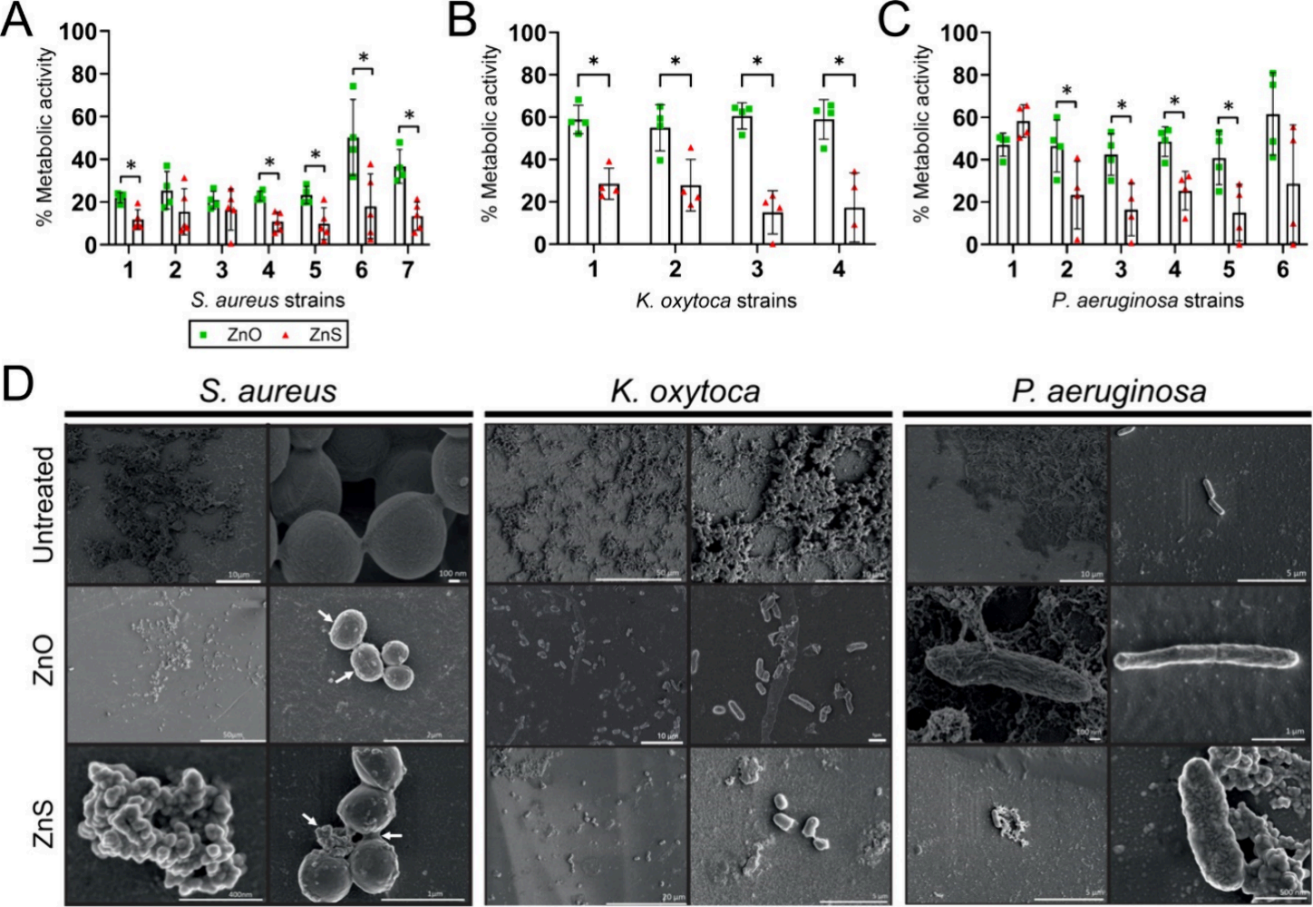
ZnS-NPs are more efficacious than ZnO-NPs in reducing the metabolic activity of mature bacterial biofilms. The percentage (%) of metabolic activity of (A) *S. aureus* (*n* = 7 strains), (B) *K. oxytoca* (*n* = 4 strains), and (C) *P. aeruginosa* (*n* = 6 strains) biofilms after treatment with ZnO- or ZnS-NPs was determined by XTT reduction assay. The biofilms were grown for 24 h at 37 °C. Bars and error bars denote the means and SDs, respectively. Each symbol represents an individual strain. Asterisks indicate *P*-value significance (* *P* < 0.05) calculated using multiple students’ *t*-test analysis. NPs-treated bacteria were compared to untreated bacteria and the percentage (%) of metabolic activity was determined using the following formula: % metabolic activity = NPs-treated bacteria/untreated bacteria x 100. (D) Scanning electron microscopy (SEM) images of untreated and ZnO- or ZnS-treated *S. aureus*, *K. oxytoca*, and *P. aeruginosa* biofilms for 24 h at 37 °C. Arrows indicate cell morphological changes.

To confirm the XTT reduction assay findings, we performed scanning electron microscopy (SEM) to observe the morphology of each mature bacterial biofilm after treatment with Zn-NPs for 24 h at 37 °C (Figure 3D). Untreated *S. aureus* biofilms show robust attachment and growth across the field (Figure 3D, upper left). A high magnification image displayed intact staphylococci clustered together in grape-like bunches (Figure 3D, upper right). ZnO-NPs-treated *S. aureus* biofilms demonstrated considerable biomass loss with individual small clusters of cells or scattered single cells and minimal extracellular matrix (Figure 3D, middle left). A cluster of four staphylococci showed two slightly elongated cells (arrows) or coccobacilli and two cocci (Figure 3D, middle right). ZnS-NPs-treated *S. aureus* biofilms exhibited a bunch of cells clustered together with minimal extracellular matrix (Figure 3D, lower left). A high magnification image showed flattered or deformed individual cells in a cluster of 4 with intertwined extracellular matrix material (arrows; Figure 3D, lower right).

Untreated *K. oxytoca* biofilms exhibited substantial bacterial aggregation in clusters and throughout the field (Figure 3D, upper left). A high magnification image shows densely aggregated bacterial clusters or biofilms (Figure 3D, upper right). ZnO (middle left) and ZnS (lower left) NPs-treated *K. oxytoca* biofilms displayed single or small clusters of dispersed bacteria throughout the fields (Figure 3D). High magnification images displayed single or small rod- (middle right) and coccobacillus- (lower right) shaped aggregates in samples treated with ZnO- and ZnS-NPs, respectively.

Lastly, untreated *P. aeruginosa* biofilms demonstrated considerable bacterial aggregation covering the upper right side of the field (Figure 3D, upper left). Closer imaging showed two rod-shaped *P. aeruginosa* cells with apparent intact and normal morphology (Figure 3D, upper right). High magnification images of ZnO-NPs-treated *P. aeruginosa* cells displayed either thickened (Figure 3D, middle left) or slimmer and enlarged (middle right) bacterial cells. Furthermore, ZnS-NPs-treated *P. aeruginosa* cells showed reduced biofilm formation (Figure 3D, lower left) and thickened and shrunk bacterial morphology with irregular surface (Figure 3D, lower right).

These findings indicate that Zn-NPs are efficacious in reducing bacterial biofilms by altering or damaging their microbial cell morphology.

### Zn-NPs Prevent Bacterial Biofilm formation

We confirmed the ability of Zn-NPs in inhibiting bacterial biofilm formation using confocal microscopy (Figure S1). We monitored *S. aureus*, *K, oxytoca*, and *P. aeruginosa* biofilm formation for 24 h in absence (untreated) or presence of ZnO- or ZnS-NPs. Since *S. aureus* was more sensitive to Zn-NPs than Gram-negative bacteria, *S. aureus* cells were treated with Zn-NPs at 0.5 mg/mL compared to 2 mg/mL for the rod-shaped bacteria. All untreated bacteria formed robust biofilms characterized by uniform cell growth and matrix distribution (Figure S1A, upper panels). Untreated *S. aureus* showed a more homogeneous (Figure S1A, upper left) and thicker biofilm (mean thickness: 220 ± 9.60 μm; Figure S1A, upper right) than cells treated with ZnO (80 ± 0.85 μm) and ZnS (44 ± 0.88 μm) NPs. Fluorescence intensity analysis revealed that untreated *S. aureus* biofilms had significantly higher fluorescence compared to ZnO (mean: 18%; *P* < 0.0001) and ZnS (mean: 8%; *P* < 0.0001) treatments (Figure S1B.) Moreover, ZnS-treated biofilms showed significantly lower fluorescence than ZnO-treated ones (*P* < 0.05). Untreated *K. oxytoca* biofilms covered the entire well bottom and displayed a thickness of 150 ± 1.89 μm. When treated with ZnO- or ZnS-NPs, biofilm thickness decreased to 72 ± 2.10 μm and 50 ± 1.65 μm, respectively. Likewise, fluorescence intensity in *K. oxytoca* biofilms was markedly reduced by both ZnO (mean: 10.47%; *P* < 0.0001) and ZnS (mean: 10.26%; *P* < 0.0001) NPs, with no significant difference between the two treatments (Figure S1C). Untreated *P. aeruginosa* biofilms reached an average thickness of 440 ± 6.25 μm, which decreased to 200 ± 4.12 μm and 100 ± 2.23 μm in the presence of ZnO- and ZnS, respectively. Similarly, fluorescence intensity was significantly reduced in treated samples (ZnO: mean 50%; ZnS: mean 11.1%; both *P* < 0.0001). ZnS-treated biofilms exhibited significantly lower fluorescence than ZnO-treated ones (*P* < 0.0001; Figure S1D). Taken together, these results demonstrate that Zn-NPs inhibit bacterial biofilm formation *in vitro.*

### Zn-NPs Alter the Expression of Genes Related to Biofilm Formation

We examined the impact of ZnO- and ZnS-NPs on bacterial expression of genes associated with biofilm formation (Table 2). We exposed *S. aureus*, *K. oxytoca*, and *P. aeruginosa* to Zn-NPs for 24 h followed by qPCR analysis. The accessory gene regulator or *agr* is important is *S. aureus* quorum sensing (QS)^37^ and biofilm formation.^38,39^ Exposure of *S. aureus* to ZnO (*agrA*, −2.08 ± 0.13; *agrC*, 1.38 ± 0.25) and ZnS (*agrA*, −2.19 ± 0.3; *agrC*, 1.08 ± 0.05) NPs resulted in moderate downregulation of *agrA* and upregulation of *agrC*. The gene *fimA* in *K. oxytoca* encodes for the type 1 fimbriae, which is an important adhesive structure that helps the bacteria attach to host cells and surfaces. Fimbriae also play a role in biofilm formation, colonization, and persistence of bacterial infections. Culture of *K. oxytoca* with ZnO- and ZnS-NPs resulted in substantial downregulation (−556.41 ± 0.15) and moderate upregulation (2.35 ± 0.13) of *fimA*, respectively. Notably, studies have shown a significant association between the presence of *fimA* and other adhesin genes (like *mrkA*, *matB*, and *pilQ*) and biofilm formation in *K. oxytoca*.^40^ Hence, we assessed *K. oxytoca*’s expression of *mrkA* after 24 h incubation with ZnO- and ZnS-NPs. ZnO- and ZnS-NPs considerably (−3147.52 ± 0.21) and moderately (−2.04 ± 0.11) downregulated the expression of *mrkA*. Additionally, *pslA* in *P. aeruginosa* is involved in exopolysaccharide biosynthesis and biofilm formation.^41^ We found that *pslA* is moderately upregulated (2.23 ± 0.17) and significantly downregulated (−2628.46 ± 0.75) by *P. aeruginosa* after incubation with ZnO- and ZnS-NPs, respectively. Similarly, *algC* in *P. aeruginosa* is a precursor for the synthesis of *psl* genes and crucial for exopolysaccharide biosynthesis that is crucial for biofilm formation.^42,43^ ZnO- and ZnS-NPs moderately upregulated (6.75 ± 0.17) and markedly downregulated (−1172.20 ± 0.54) the expression of *algC*. These findings suggest that bacterial exposure to Zn-NPs alter the expression of genes associated with biofilm formation.

**Table 2:** Quantification of Gene Expression Modulation by ZnO- and ZnS-NPs in *S. aureus*, *K. oxytoca*, and *P. aeruginosa* Using RT-qPCR

*S. aureus*	%	SD	*K. oxytoca*	%	SD	*P. aeruginosa*	%	SD
*agr*A ZnO	–2.08^a^	0.13	*mrk*A ZnO	–3147.5^a^	0.21	*psl*A ZnO	2.23	0.16
*agr*A ZnS	–2.19^a^	0.3	*mrk*A ZnS	–2.04^a^	0.11	*psl*A ZnS	–2628.5	0.75
*agr*C ZnO	1.38	0.25	*fim*A ZnO	–556.41^a^	0.15	*alg*C ZnO	6.75	0.17
*agr*C ZnS	1.08	0.05	*fim*A ZnS	2.35	0.13	*alg*C ZnS	–1172.2	0.54

a Negative signal (−) indicates lower expression of the target genes. Values are presented as mean ± standard deviation (SD) of three independent experiments (*n* = 3). Target genes were normalized using the 16S rRNA gene as an internal control.

### Zn-NPs Do Not Stimulate Cutaneous Wound Healing but Reduce Microbial Load

We used a validated mouse model of excisional cutaneous wounds and microbial infection^26–28^ with minor modifications to assess the impact of Zn-NPs on these processes. First, we evaluated skin wound healing after infection of Balb/c mice (*n* = 4 mice per group) with 10^7^ *S. aureus* 553838 or *P. aeruginosa* MRSN 5519 cells at 3-days postwounding (dpw; Figure S2). These clinical strains were selected because they were isolated from human cutaneous wounds. Mice were treated twice daily with 5 mg/mL of either ZnO- or ZnS-NPs. Uninfected and untreated or 1% dimethyl sulfoxide (DMSO; NP vehicle) and infected mice were used as controls. We did not observe differences in wound healing in the infected mice with *S. aureus* (Figure S2A,C) or *P. aeruginosa* (Figure S2B,D) treated with ZnO- or ZnS-NPs.

Then, we determined the effect of ZnO- or ZnS-NPs on *S. aureus* (Figure 4A) and *P. aeruginosa* (Figure 4B) cutaneous tissue load using colony forming units (*n* = 4 mice per group; CFU; Figure 4A,C) counts and histology (Figure 4B,D). Wounded tissue excised from the back of untreated (mean: 1 × 10^10^ CFU/g tissue) and 1% DMSO (mean: 9.58 × 10^9^ CFU/g tissue) mice had high and similar staphylococcal burden (Figure 4A). Untreated and 1% DMSO wounds showed significantly higher CFU than wounds treated with ZnO (mean: 6.38 × 10^8^ CFU/g tissue; *P* < 0.0001) and ZnS (mean: 8.06 × 10^8^ CFU/g tissue; *P* < 0.0001) NPs. Although wounds removed from ZnS-NPs-treated mice had higher CFU counts relative to those from ZnO-NPs-treated animals, these differences were not statistically significant (Figure 4A). Hematoxylin and eosin (H&E) straining for general tissue morphology and Gram staining for bacteria were performed to understand early infection development and the impact of Zn-NPs treatment in wound microbial burden (Figure 4B,D). Uninfected wounds exhibited an inflammatory response in the dermis and hypodermis (20X; white arrows; Figure 4B). Also, an absence of infection was confirmed by Gram staining (Figure 4B**,** inset). Untreated wounded tissue showed substantial accumulation of staphylococci on the surface of the lesion (20X) that could be appreciated at higher magnification (63X; red arrowheads) and in the Gram staining (Figure 4B**,** inset). Inflammation in untreated and infected tissue with *S. aureus* is visible in the dermis and hypodermis (20X; white arrows). Wounds treated only with 1% DMSO also displayed high bacterial burden on the surface (20X and 63X), but without visible inflammatory response. ZnO- and ZnS-NPs treated wounds also had visible staphylococcal burden on the surface and minimal inflammation (Figure 4B)**.**

**Figure 4:**
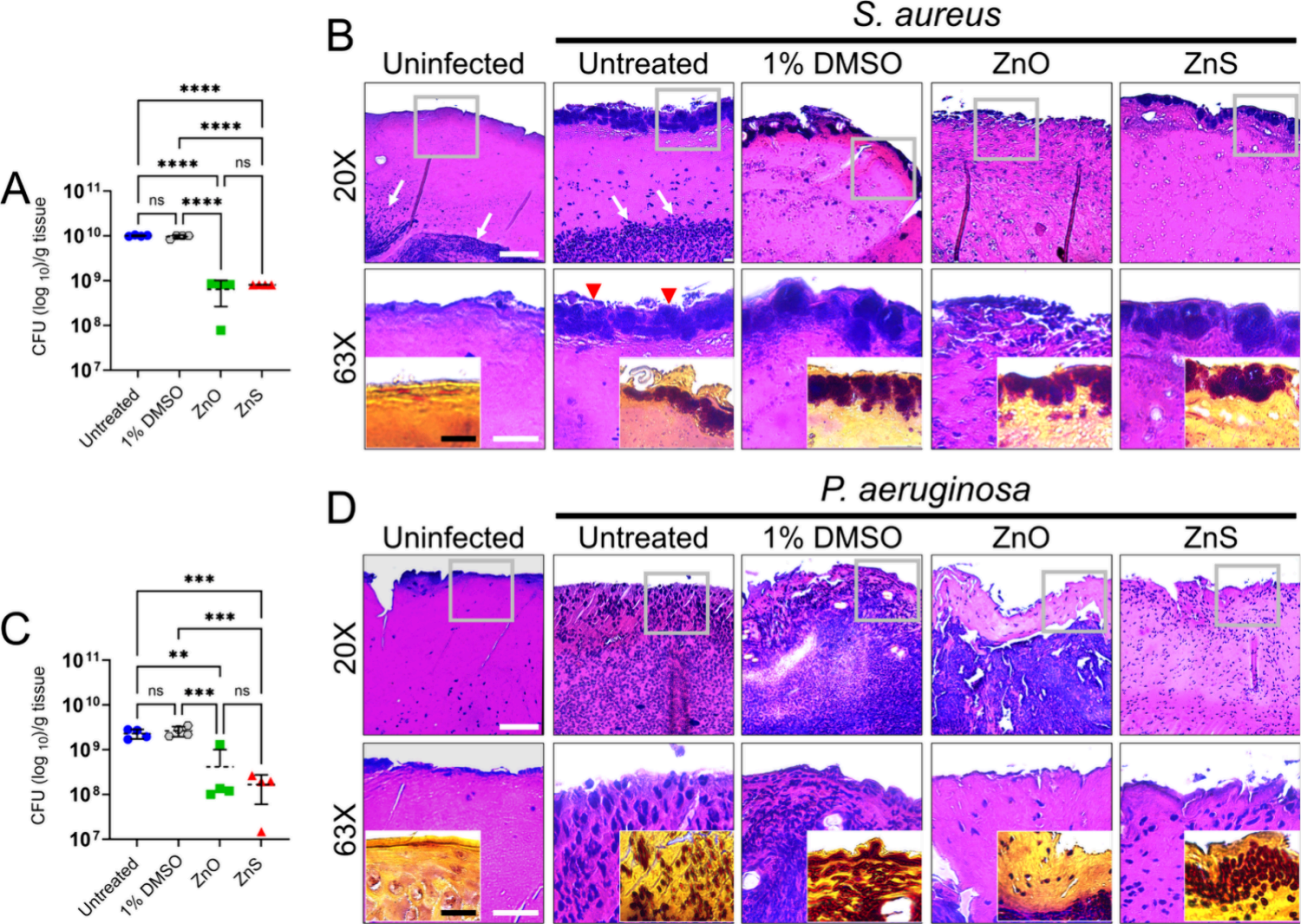
Zn-NPs reduce wound bacterial burden. (A) *S. aureus* 553838 and (C) *P. aeruginosa* MRSN 5519 colony forming units (CFU) per gram of tissue were determined for each treatment at 3-dpw. Mice were infected with 10^7^ bacteria. For A and C, dashed lines and error bars denote the means and SDs, respectively. Each symbol represents an individual wound (*n* = 4 per group). Asterisks denote *P*-value significance (**P < 0.01, ***P < 0.001, and ****P < 0.0001) calculated using ANOVA and adjusted by use of Tukey’s posthoc analysis. ns denotes comparisons which are not statistically significant. Histological analysis of wounds (*n* = 4) from uninfected and (B) *S. aureus*- and (D) *P. aeruginosa*-infected Balb/c mice at 3-dpw. White arrows and red arrowheads indicate inflammation and bacterial load, respectively. Untreated and treated wounds with 1% DMSO, ZnO- or ZnS [dose: 5 mg/mL twice daily] were imaged using light microscopy (20X and 63× magnification) and pathologically analyzed. Representative H&E-stained sections of the skin lesions are shown with the insets representing Gram staining for bacteria (*S. aureus* and *P. aeruginosa* are shown in purple and red-pink, respectively). Gray squares in the low magnification images indicate the region that was magnified in the images taken at 63X. Scale bars = 100 μm.

Wounds infected with *P. aeruginosa* that did not receive treatment (mean: 2.3 × 10^9^ CFU/g tissue) or received 1% DMSO (mean: 2.64 × 10^9^ CFU/g tissue) treatment showed similarly high bacterial load (Figure 4C). Untreated and 1% DMSO wounds showed significantly higher CFU than wounds treated with ZnO (mean: 4.14 × 10^8^ CFU/g tissue; untreated, *P* < 0.01; 1% DMSO, *P* < 0.001) and ZnS (mean: 1.67 × 10^8^ CFU/g tissue; *P* < 0.001) NPs. There were no differences in wounded tissue bacterial burden between the Zn-NPs treatments (Figure 4C). H&E staining of uninfected wound tissue demonstrated no inflammation (20X and 63X) or bacterial presence (inset; Figure 4D). Untreated and 1% DMSO-treated tissue exhibited considerable and uniformed inflammation across the skin layers (20X and 63X), which was associated with a uniform distribution of *P. aeruginosa* cells (Gram staining; Figure 4D**,** inset). Tissue treated with 1% DMSO exhibited more acute inflammation, characterized by substantial immune cell infiltration than untreated tissues, likely related to the differential bacterial accumulation in tissue as the vehicle- treated tissue exhibited a more compact microbial tissue load. Interestingly, wounded and infected tissue treated with ZnO-NPs exhibited minimal inflammation in the epidermis or tissue surface (20X and 63X) and acute inflammation in the dermis and hypodermis (20X; Figure 4D). Gram staining also shows major bacterial accumulation in dermal and hypodermal tissue compared to the tissue surface or epidermis (Figure 4D**,** inset). In contrast, wounded tissue treated with ZnS-NPs showed reduced inflammation across tissue with scattered immune cell infiltration (20X and 63X) and higher microbial load on the tissue surface (Figure 4D**,** inset). These data showed that Zn-NPs treatment of infected cutaneous wounds does not affect early healing but reduce tissue *S. aureus* and *P. aeruginosa* load.

### Zn-NPs Protect Cutaneous Wound Tissue Collagen from Bacterial Degradation

Collagen is a structural skin protein that can get degraded during infection. We investigated the impact of ZnO- and ZnS-NPs on collagen protection after early *S. aureus* 553838 and *P. aeruginosa* MRSN 5519 infection (3-dpw) using trichrome staining and light microscopy (Figure 5). We utilized NIH ImageJ software to analyze each image and determine the intensity of collagen, which stains blue. Uninfected wounded tissue showed high collagen intensity (Figure 5A–D). Untreated and 1% DMSO-treated tissue and infected with *S. aureus* (Figure 5A,B) or *P. aeruginosa* (Figure 5C,D) had a significant reduction in collagen intensity relative to uninfected (*P* < 0.0001 and *P* < 0.0001) and ZnO (*S. aureus*, *P* < 0.0001 and *P* < 0.01; *P. aeruginosa*, *P* < 0.0001 and *P* < 0.0001) or ZnS (*S. aureus*, *P* < 0.0001 and *P* < 0.01; *P. aeruginosa*, *P* < 0.0001 and *P* < 0.0001) NPs treated and infected wounds (Figure 5B,D). Uninfected tissue demonstrated higher collagen intensity or preservation than ZnO (*S. aureus*, *P* < 0.05; *P. aeruginosa*, *P* < 0.0001) and ZnS (*S. aureus*, *P* < 0.05; *P. aeruginosa*, *P* < 0.0001) NPs treated tissue infected with bacteria. Nevertheless, there was no difference in wound tissue collagen intensity between Zn-NPs treated tissue and infected with either bacterium (Figure 5B,D). Our results demonstrate that cutaneous wounded tissue treated with Zn-NPs preserves skin tissue collagen content early during infection.

**Figure 5:**
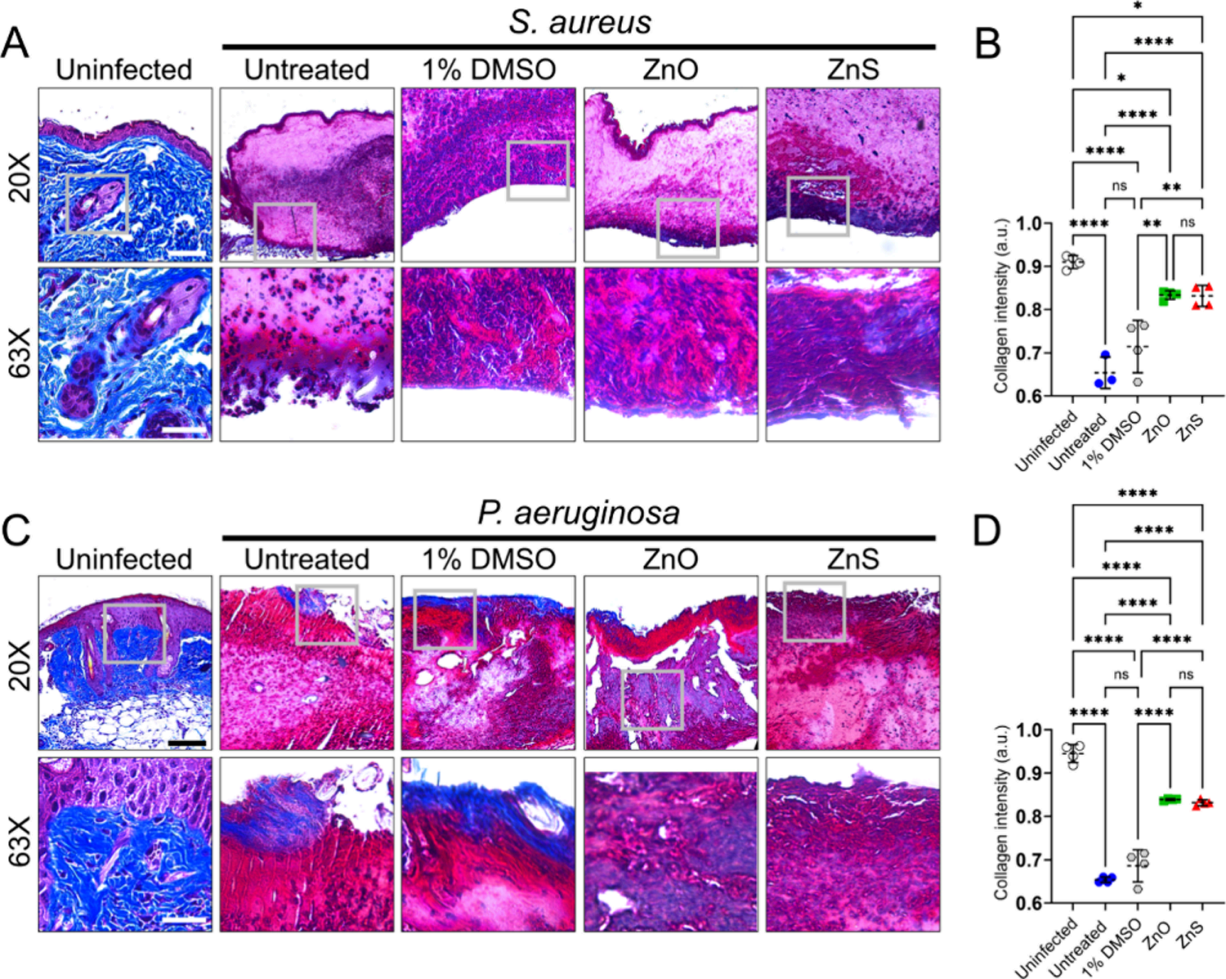
Zn-NPs maintain collagen in skin lesions of Balb/c mice infected with bacteria. Histological analysis of wounds (*n* = 4) from uninfected and (A) *S. aureus* 553838 and (C) *P. aeruginosa* MRSN 5519 infected Balb/c mice at 3-dpw. Untreated and treated wounds with 1% DMSO, ZnO- or ZnS [dose: 5 mg/mL twice daily] were imaged using light microscopy (20X and 63× magnification) and analyzed. Representative trichrome-stained sections of the skin lesions are shown. The blue stain indicates collagen. Scale bars = 100 μm. Quantitative measurement of collagen intensity in the wounds of (B) *S. aureus*- and (D) *P. aeruginosa*-infected mice, untreated or treated with 1% DMSO, ZnO- or ZnS [dose: 5 mg/mL twice daily]. Four wounds per group were analyzed. For B and D, dashed lines are the averages of 10 fields per wound, and error bars denote SDs. Asterisks denote *P*-value significance (*P < 0.05, **P < 0.01, and ****P < 0.0001) calculated using ANOVA and adjusted by use of the Tukey’s posthoc analysis. ns denotes comparisons which are not statistically significant.

## Discussion

In this study, we successfully prepared and tested the effectiveness of described formulations of ZnO^44^ and ZnS^23^ NPs against medically significant bacteria due to their capability to form biofilms and develop resistance to commonly prescribed antibiotics. The results from the MIC test suggest that the planktonic cells of a single Gram-negative *K. oxytoca* strain exhibit greater sensitivity to Zn-NPs in comparison to the Gram-positive *S. aureus* and Gram-negative *P. aeruginosa* strains. In this regard, *K. oxytoca* has shown variability in susceptibility or resistance to antibiotics,^14^ depending on individual strain expression of efflux pumps,^45^ production of β-lactamases and/or carbapenemase,^46,47^ and their acquisition of resistance genes via horizontal transfer.^48^ Thus, continuous monitoring of *K. oxytoca* antimicrobial resistance patterns is essential for effective hospital infection control and patient care. In addition, due to the close association of *S. aureus* and *P. aeruginosa* with humans, it is possible that the myriads of virulence factors produced by these bacteria provide multiple mechanisms of resistance to antimicrobials including Zn-NPs. For example, *P. aeruginosa* generates pyocyanin, which helps it defend against NPs by neutralizing ions that are produced or released.^49^ In addition, the *P. aeruginosa* strain CCM 3955 produces an overabundance of a flagellin matrix that leads to the clustering of silver NPs to prevent direct interaction, thus exhibiting bactericidal properties.^50^ *S. aureus* can develop resistance to silver NPs through mechanisms like increased biofilm production and NP aggregation.^51^ In fact, *S. aureus* biofilm-derived cells have shown more resistance to nitric oxide NPs than their planktonic counterparts.^52^ The ability of bacteria with pathogenic potential to modulate their virulence factors provides an advantage during their interactions with the human host, plays a crucial role in the development of antimicrobial resistance, and should be constantly monitored and investigated to understand the mechanisms of resistance.

The results from the XTT reduction assay and SEM/confocal microscopy showed that biofilm formation was significantly reduced when bacteria were cultured with Zn-NPs, confirming findings from MIC assays. Interestingly, most of the isolates for each bacterial species showed reduced metabolic activity, an established measurement of biofilm formation, when exposed to ZnS than ZnO-NPs. It is plausible that Zn-NPs disrupt cell-to-cell interactions by modifying the surface negative charge or zeta potential, thereby preventing adhesion to surfaces. For instance, ZnO-NPs decrease the hydrophobic characteristics of *S. aureus* and *P. aeruginosa* cell surfaces, which may account for the observed decrease in their ability to form biofilms.^53^ Likewise, Zn-NPs might influence gene expression in biofilm formation, particularly genes linked to adhesion structures,^54^ as well as QS^55^ and the synthesis of the extracellular matrix.^54^ Therefore, we measured the expression of genes involved in adhesion and/or biofilm formation in the tested bacteria after incubation with Zn-NPs. Treatment of *S. aureus* with ZnO- and ZnS-NPs downregulated *agrA* and upregulated of *agrC*, which are critical for QS^37^ and biofilm formation.^38,39^ Also, on average, *S. aureus* isolates showed lower metabolic activity than *K. oxytoca* and *P. aeruginosa* isolates. We have previously shown that *S. aureus* was more susceptible to Zn-NPs than *K. oxytoca* and *P. aeruginosa*.^23^ ZnO-NPs substantially downregulated *K. oxytoca*’s *fimA* and *mrkA*, two related adhesins, providing evidence for the reduced biofilm formation observed in this species. Another interesting observation is that even when ZnS-NPs reduced significantly the metabolic activity of *K. oxytoca* strains, it is conceivable that their effects are not on the bacterial adhesion or biofilm formation abilities given their moderate effect on *fimA* and *mrkA* expression, but on disrupting other cellular processes or components. Zn-NPs can inhibit bacterial growth by promoting the generation of reactive oxygen species (ROS) and the release of Zn^2+^ ions. ZnS hampers bacterial growth and prevents biofilm formation by increasing ROS production, all of which can damage cellular components like DNA, proteins, and lipids through oxidative stress.^56^ Moreover, Zn^2+^ ions can be generated after the dissolution of ZnS-NPs. These Zn^2+^ ions extend the lag phase of bacterial growth and disrupt various biochemical processes, including glycolysis, transport of protons across the membrane, acid tolerance, and QS.^57^ Furthermore, ZnS-NPs significantly downregulated the expression of *pslA* and *algC* in *P. aeruginosa*, both critical for biofilm formation and exopolymeric matrix synthesis, which explains the reduced metabolic activity and impaired biofilm formation. A previous study has shown that Zn-NPs have QS inhibitory effects and consequently impair the development of biofilms.^58^ Similarly, Zn-NPs significantly reduce *P. aeruginosa*’s protease activity, elastase activity, and pyocyanin production.^58^ Understanding these mechanisms is essential for further insight on how ZnS-NPs eradicate bacteria. Hence, it is important to highlight that most of the published data, including ours,^23^ indicate that Gram-positive bacteria show greater sensitivity to Zn-NPs relative to their Gram-negative counterparts, which may be due to interference with bacterial structural integrity, harm caused by ROS, or the suppression of transcription for genes that confer resistance to oxidative stress.^59^

We did not observe differences in early wound healing between Zn-NPs-treated and control mice. It is possible that our Zn-NP formulations require additional modifications to make them efficacious on enhancing wound healing. Others have shown that Zn-NPs promote neutrophils and macrophages recruitment.^60^ However, it is still controversial whether the participation of innate immune cells on wound healing is positive or negative and potentially varies from person to person.^61,62^ In contrast, we demonstrated that Zn-NPs reduce wound bacterial burden in tissues infected with *S. aureus* or *P. aeruginosa*. Notably, each bacterial species displayed different skin infection progression. For example, Zn-NPs treated wounds infected with *S. aureus* evinced visible staphylococcal burden on the cutaneous surface (epidermis) and minimal inflammation at 7-dpw, suggesting that microbial elimination is likely due to the bactericidal effects of Zn-NPs. However, *P. aeruginosa* infection was deeper (e.g., dermis and hypodermis) in the cutaneous tissue with colonization showing mixed inflammatory responses depending on the Zn-NPs treatment. ZnO-NPs-treated tissue exhibited minimal epidermal inflammation and severe dermal inflammation. In addition, substantial microbial growth in dermal and hypodermal tissue was observed relative to those observed in epidermal tissue. Perhaps, ZnO-NPs requires high activation of inflammatory cells to eliminate *P. aeruginosa* cells. In contrast, ZnS-NPs-treated wounds demonstrated reduced inflammation across tissue with scattered immune cell infiltration and higher bacterial burden on the epidermal tissue. This postulate is supported by the XTT reduction assay data, which demonstrated that most *P. aeruginosa* isolates treated with ZnS-NPs had significantly lower metabolic activity than those treated with ZnO-NPs. CuInS/ZnS quantum dots promote ROS production and reduce tissue adhesion and biofilm formation,^63^ thus, eliminating the need for a strong inflammatory response. Regardless of the mechanism by which ZnO- and ZnS-NPs stimulate or abrogate host skin inflammation, Zn-based NPs similarly reduce microbial load compared to the control groups.

We showed that Zn-NPs protect cutaneous wound tissue collagen from bacterial degradation, and this can be crucial for the treatment of chronic wounds. *S. aureus* ^64^ and *P. aeruginosa* ^65^ produce proteases that can directly degrade skin collagen and other components of the extracellular matrix, weakening the tissue and delaying repair. Zn positively influences biological processes including collagen synthesis and the development of blood vessels.^66,67^ A chondroitin ZnS complex has demonstrated anti-*S. aureus* and -*Escherichia coli* activity in wounds.^68^ In addition, this complex promoted healing after stimulating the production of collagen, vascular endothelial growth factor, and fibroblast growth factor beta and reducing the synthesis of inflammatory cytokines.^68^ Thus, it is likely that ZnS-NPs prevent collagen degradation by killing bacteria and minimizing inflammation, which is demonstrated by the H&E staining results. For instance, neutropenic mice exhibit elevated collagen expression and levels in their wounded skin tissues.^69^ Neutrophils can also generate collagenases that break down collagen during the initial stages of a wound and their reduction may directly lead to a greater overall collagen content in wounds.^70^ Studies focusing on chronic infections and the modulation of inflammatory cellular and molecular responses by Zn-NPs are necessary to understand the mechanisms of skin tissue integrity maintenance.

Zn-NPs are a promising antimicrobial strategy to combat drug-resistant bacteria. Although the current preparations of Zn-NPs did not show wound healing in mice, the observation of early (3-dpw) collagen preservation during infection suggests their potential involvement in cutaneous tissue integrity and highlights their therapeutic potential for the treatment of chronic infections. In contrast, analyses at 7- and 21-dpw did not reveal differences in wound bacterial burden or collagen preservation between Zn-NPs-treated and control groups (data not shown). Since Zn-NPs were applied in saline without a vehicle to enhance absorption (e.g., water-based gels), it is possible that their collagen preservation impact on later stages of wound healing was minimal due to reduced NP penetration through the murine skin layers.^71^ This possibility can be easily tested in the future as this technology is still in development. Like in medicine, biofilm-related contamination is also problematic for the food industry resulting in significant economic loss every year.^72^ For instance, in today’s globalized market, the transportation and consumption of fresh food is of upmost priority, thus, food spoilage linked to bacteria may compromise food safety. Bacteria can contaminate food and causes episodes of food poisoning in humans due to toxin production. The CDC estimates that *S. aureus* causes ∼ 250,000 illnesses per year in the U.S.,^73^ although this approximation may be higher since *S. aureus*-related sporadic food-borne disease is not reportable.^74^ Hence, Zn-NPs can be utilized in surface coating applications to inhibit biofilm development, such as on catheters in the medical field, serve as antimicrobial agents, or be applied in the food industry to minimize areas of nondeposition. The observed efficacy of Zn-NPs against drug-resistant bacteria makes them a potential antibacterial tool. Although Zn-NP treatment did not accelerate wound closure in mice, their ability to preserve collagen during infection highlights a protective effect on skin tissue integrity. Moreover, the observed inhibition of biofilm formation supports potential applications in medical devices and food-contact surfaces to reduce bacterial colonization and contamination.

Despite their antimicrobial effectiveness, the Zn-NPs used in this study have drawbacks, including our incomplete understanding of their specific antimicrobial mechanism of action, solubility and aggregation issues, technical challenges associated with cytotoxicity measurements, limited availability of pharmacokinetics *in vivo* and the restricted translational relevance of our *in vivo* wound healing model to clinical settings. Addressing these challenges through mechanistic studies, formulation optimization, and more clinically relevant models will be essential to fully realize the therapeutic potential of Zn-NPs in combating multidrug-resistant wound infections.

## Material and Methods

### Bacteria

*S. aureus* (Gram-positive; ATCC 25923), *K. oxytoca* (Gram-negative; ATCC 13182), and *P. aeruginosa* (Gram-negative; ATCC 27853) strains were used in all the *in vitro* experiments and grown in Muller-Hinton broth (Remel) or agar (Oxoid). Clinical strains *S. aureus* 553838 and *P. aeruginosa* MRSN 5519 were isolated from human skin wounds and used in the *in vivo* experiments. Microbial suspensions were prepared from a single colony grown overnight in fresh medium at 37 °C and adjusted to 0.1 of optical density (625 nm) equivalent to 10^8^ colony-forming units per milliliter (CFU/mL) using a spectrophotometer and according to the protocol of the Clinical and Laboratory Standards Institute (CLSI).^75^ In addition to the standard ATCC strains, multiple strains of each bacterial species (Table 1) were used in the biofilm experiments shown in Figure 3.

### Synthesis of Zn-NPs

#### ZnO

The synthesis of ZnO-NPs was performed by the sol–gel method as described^29^ with a few modifications. Initially, 1.12 g of Zn acetate dihydrate (Sigma-Aldrich, 98%) were added to 50 mL of ethanol (Dinâmica). The solution was heated under reflux at 98 °C for 3 h. The Zn acetate precursor solution was cooled to room temperature (RT), diluted with ethanol to 100 mL and stored at 4 °C. The final concentration of Zn acetate precursor was 0.05 mol/L. The preparation of ZnO colloidal suspension was carried out by adding 0.214 g of lithium hydroxide (Chemicals). Then, the reaction was carried out in ultrasound at 50 °C for 1 h. The surface of the ZnO-NPs was modified using a (3-Glycidyloxypropyl) trimethoxy silane (GPTMS; Sigma-Aldrich, 98%) modifier. 0.428 g of lithium hydroxide and 2.25 mL of GPTMS were added. Then, the reaction was carried out under ultrasound for 30 min at 35 °C. Finally, the solution was centrifuged, the white powder dried in an oven and then dispersed for characterization and further testing.

#### ZnS

The synthesis of ZnS-NPs was performed by the sol–gel method as described^30^ with a few modifications. Solution 1 was prepared using the following steps: 1.12 g of Zn acetate dihydrate were added to 50 mL of ethanol. Then, the solution was heated under reflux at 98 °C for 3 h. The Zn acetate precursor solution was cooled to RT, diluted with ethanol to 100 mL and stored at 4 °C. The final concentration of the Zn acetate precursor solution was 0.05 mol/L. Solution 2 was prepared using the following steps: 0.383 g of thiacetamide (Sigma-Aldrich, 98%) was added in 100 mL of ethanol at RT to a final concentration of 0.005 mol/L. Finally, the reaction step was prepared by heating 100 mL of solution 1 at 60 °C in a round-bottom flask. Then, solution 2 was added to the flask and the reaction was carried out for 12 min stirring the mixture at 400 rpm. The mixture was cooled, precipitated, and centrifuged several times with the addition of heptane (Exodus Científica) in a 1:4 ratio. The white powder formed was characterized and used for further testing.

#### Zn-NP Physical Properties

We characterized the structural properties of ZnO- and ZnS-NPs by UV–vis absorption spectrum, X-ray diffraction, and Zeta potential measurements. The UV–vis absorption spectrum was performed using a Cary Win 4000 spectrophotometer (Agilent) and collected between 200 to 800 nm. X-ray diffraction analysis was performed using a D5000 diffractometer (Siemens) with Cu Kα radiation (λ = 1.5418A). Data were collected at RT over an angle range of 5–80° 2θ. The Zeta potential of Zn-NPs was measured using a Zetasizer Nanoseries instrument (Malvern). Samples in triplicate were dispersed into an aqueous medium with 0.5% dimethylsulfoxide (DMSO) at 25 °C.

### Determination of the MIC

To determine the MIC of several strains of each bacterial species (Table 1) to Zn-NPs, we used a microdilution method in 96-microtiter well plates according to the M7-A10 protocol of the CLSI^75^ that was modified by using Mueller-Hinton medium. The Zn-NP concentration range selected for testing were 10 to 0.02 mg/mL and 2.5 to 0.1 mg/mL for Gram-negative and Gram-positive bacteria, respectively. A 100-μL suspension of Mueller-Hinton broth with 10^6^ CFU was added per well. Medium without bacteria or with bacteria but without Zn-NPs were used as negative and positive controls, respectively. The microtiter plates were incubated with shaking at 150 rpm in a Bioscreen C at 37 °C for 24 h. The absorbance was measured every 20 min at 600 nm. The MIC_50_ for each bacterium was calculated by determining the Zn-NP concentration that reduced 50% of the microbial population (absorbance) relative the control.

### Biofilm Formation

Two hundred microliters of a suspension with 10^5^ bacteria in Muller-Hinton broth (Sigma) alone or with Zn-NPs (0.5 mg/mL, *S. aureus* or 2 mg/mL, *K. oxytoca* or *P. aeruginosa*) were added to individual wells of polystyrene 96-well microtiter plates. As a control, the initial inoculum of each bacterial species was serially diluted and plated on Muller-Hinton agar to corroborate having the same number of microbial cells per condition. The plates were incubated at 37 °C in a 5% CO_2_ aerobic atmosphere, and biofilms were formed over 24 h. After incubation, the medium was gently aspirated, and biofilms were gently washed three times with 200 μL of PBS to remove nonadhered bacteria. Bacteria that remained attached to the plastic surface were considered true biofilms. Biofilm formation was verified by XTT reduction assay and SEM/confocal microscopy.

### XTT Reduction Assay

The XTT reduction assay was performed to assess the efficacy of Zn-NPs in reducing bacterial biofilms metabolic activity from diverse strains (Table 1). Aliquots of 50 μL of XTT salt solution (1 mg/mL in PBS) and 4 μL of menadione solution (1 mM in acetone; Sigma) were added to each well of a 96-well microtiter plate. Microtiter plates were then incubated at 37 °C for 5 h. The electron transport system in the cellular membrane of live bacteria reduces the XTT tetrazolium salt to XTT formazan, resulting in a colorimetric change, which was measured in a microtiter reader at 492 nm. Microtiter wells containing only medium without bacterial cells were used as negative controls.

### SEM

SEM analyses were performed to characterize bacterial morphology after treatment with ZnO- or ZnS. After dehydration, the samples were placed in a vacuum desiccator until analysis. Each sample was coated with gold by sputtering for 20 s under pressure of 2 × 10–1 mbar and examined in the high-resolution SEM JEOL JSM-7500F (Jeol USA) with PC-SEM v 2,1,0,3 operating software, equipped with secondary electron backscattered detectors.

### Quantitative RT-PCR (qPCR)

Triplicate cultures of each bacterium were grown in absence or presence of Zn-NPs in Müeller-Hinton broth at 37 °C, 240 rpm, and harvested after 24 h. The Zn-NPs concentrations were selected based on each bacterium MIC_50_. Total RNA was extracted using ferromagnetic extraction kit (Abbott Molecular Biology) following the manufacturer’s instructions. cDNA was synthesized using the iScript reverse transcriptase kit (Bio-Rad). The primers used for expression analysis are listed in Table 3. qPCR was carried out using POWER SYBR Green Supermix on a StepOnePlus thermocycler (Applied Biosystems), with the 16S rRNA gene as the reference. Reactions were set up according to the manufacturer’s protocols using 500 nM primers and 2 μL of the cDNA template (diluted 1:10). Relative expression was determined using the comparative cycle threshold (ΔΔCT) method.^76^ The cycling conditions used were as follows: amplification stage – 95 °C for 10 min and then 40 amplification cycles of 95 °C for 30 s, 60 °C for 1 min and 72 °C for 30 s; melting curve stage – 95 °C for 15 s, 60 °C for 1 min and 95 °C for 15 s. No-template and no-reverse transcriptase reactions served as the negative controls. All reactions were carried out in triplicate, using cDNA derived from triplicate cultures.

**Table 3:** Target Genes and Oligonucleotides for RTqPCR Amplification

bacteria	gene	primer	sequence 5′-3′	temperature (°C)
S. aureus	*ag*rA	*agr*A-F	GCAGTAATTCAGTGTATGTTCA	60
		*agr*A-R	TATGGCGATTGACGACAA	60
	*ag*rC	*agr*C–F	GCAGTATTGGTATTATTCTTGA	60
		*agr*C-R	TGCGTGGTATATCATCAG	60
K. oxytoca	*mrk*A	*mrk*A-F	CTGGCCGGCGCTACTGCTAAG	61
		*mrk*A-R	CACCCGGGATGATTTTGTTGG	61
	*fim*A	*fim*A-F	GCACCGCGATTGACAGC	61
		*fim*A-R	CGAAGGTTGCGCCATCCAG	61
P. aeruginosa	*psl*A	*psl*A-F	TACCGGGCCTGGATGA	60
		*psl*A-R	CGGCAGCGAGTTGTAGTT	60
	*alg*C	*algC*-F	CCTACCCCGGTGCTGTACTA	58
		*algC*-R	GATGCCAGGTCGTTTTTCTC	58
16S RNA		16S–F	TGATCCTGGCTCAGGATGA	
		16S-R	TTCGCTCGACTTGCATGTA	

### Ethics Statement

All animal studies were conducted according to the experimental practices and standards approved by the Institutional Animal Care and Use Committee (IACUC) at the University of Florida (Protocol #: IACUC 202200000675).

### *In Vivo* Wound Model and Zn-NPs Treatment

To investigate the antimicrobial efficacy of Zn-NPs in cutaneous wounds infected with *S. aureus* 553838 or *P. aeruginosa* MRSN 5519 strains, female Balb/c mice (6 to 8 weeks old; Envigo) were used. The animals were anesthetized with 100 mg/kg ketamine and 10 mg/kg xylazine, their back fur was shaved, and their skin was disinfected with iodine. Circular wounds of 5 mm in diameter were created in the center of the animals’ backs using surgical punctures. A 50-μL suspension containing 10^7^ bacteria in saline was inoculated into each wound. One day postinfection, a suspension containing 5 mg/mL of Zn-NPs dissolved in sterile saline supplemented with 1% DMSO was topically applied to each wound twice daily. Uninfected and infected mice untreated or treated with 1% DMSO (vehicle) were used as controls. Wounds were photographed daily and blindly measured by two independent investigators. Three days postinfection, the animals were euthanized, and the wounds were removed.

### CFU Determinations

Wounded tissues were weighed, homogenized in sterile saline, and serially diluted. Samples were plated on Müller-Hinton agar and bacterial colonies were determined. The results were normalized by tissue weights and reported as CFU (log 10)/gram of tissue.

### Histological Examinations

Wounded tissues were fixed in 10% formalin for 24 h, processed, and embedded in paraffin. Four-μm vertical sections were fixed to glass slides and subjected to Hematoxylin and Eosin (H&E), Gram, and Masson’s trichrome staining to examine tissue morphology, bacteria, and collagen deposition, respectively. Slides were examined by light microscopy.

### Collagen Deposition Assessment

Collagen deposition was measured by calculating the trichrome blue color intensity in wounded tissues using the NIH ImageJ color deconvolution tool software (version 1.53q) as described.^26–28^

### Statistical Analysis

All data were subjected to statistical analysis using Prism 10.1.3 (GraphPad). *P* values for multiple comparisons were calculated by analysis of variance (ANOVA) and adjusted using Tukey’s multiple comparison test. The multiple student’s *t*-test was used to analyze individual comparisons. *P* values of <0.05 were considered significant.

## Supplementary Material



## Notes

Materials and methods: confocal microscopy and wound healing *in vivo*; Results: SFigure 1, Zn-NPs inhibit bacterial biofilm formation; SFigure 2, Zn-NPs do not promote cutaneous wound healing (PDF)
