# Supplementary material for: Zinc-Based Nanoparticles Reduce the Bacterial Burden and Protect Collagen in a Mouse Cutaneous Wound Model
Source: ACS Omega. 2026 Jan 24;11(5):7115–28. doi: 10.1021/acsomega.5c06287 (PMC12902840; doi:10.1021/acsomega.5c06287)
Supplement: Supplementary file 1 [file ao5c06287_si_001.pdf]

Supporting Information

Manuscript: "Zinc-based nanoparticles reduce the bacterial burden and protect collagen in a mouse cutaneous wound model" (ao-#2025-062878)

Authors: Rafael Bianchini Fulindi, Thulio Wliandon Lemos Barbosa, Vanessa Enriquez, Claudia L. Charles-Niño, Natália Galvão de Freitas, Mariana Picchi Salto, Leila Aparecida Chiavacci, Sebastião Pratavieira, João Pessoa Araújo Junior, and Luis R. Martinez

Corresponding author: Paulo Inácio da Costa, Ph.D.

SUPPLEMENTARY MATERIALS AND METHODS

Confocal microscopy. The architecture of biofilms was examined as described.[23] Briefly, the biofilm structure was examined using the Live/Dead viability kit (Invitrogen) and confocal microscopy. Biofilms were grown for 24 h on 35 mm glass-bottom plates, alone or with Zn-NPs at the MIC_{50} for each strain. Samples were rinsed with PBS, stained with SYTO9 (6 µL in 2 mL dH_2O) for 30 min at RT in the dark, and rinsed again. Imaging was performed with a Zeiss LSM 780 inverted confocal microscope. X-Y optical sections (1.175 µm) were collected through the biofilm using a 63× objective lens. Fluorescence intensity/μm^2 was quantified in regions of interest of 100 µm × 100 µm using ImageJ V5.3, and Z-stack images were deconvoluted with Zeiss Zen Lite software.

Wound healing *in vivo*. Balb/c mice were infected with *S. aureus* 553838 or *P. aeruginosa* MRSN 5519 (10^7 CFU per wound). Wounds were untreated or treated twice daily for 3-days post-wounding (dpw) with 1% DMSO (vehicle) or 5 mg/mL ZnO- or ZnS nanoparticles. Wound images were acquired at indicated time points, and wound closure was measured blindly with calipers by two independent investigators. Data are presented as mean ± SD (n = 4 mice per group; a single wound per mouse). Statistical significance was calculated using ANOVA with Tukey's post-hoc test.

SUPPLEMENTARY FIGURES (SFig.)

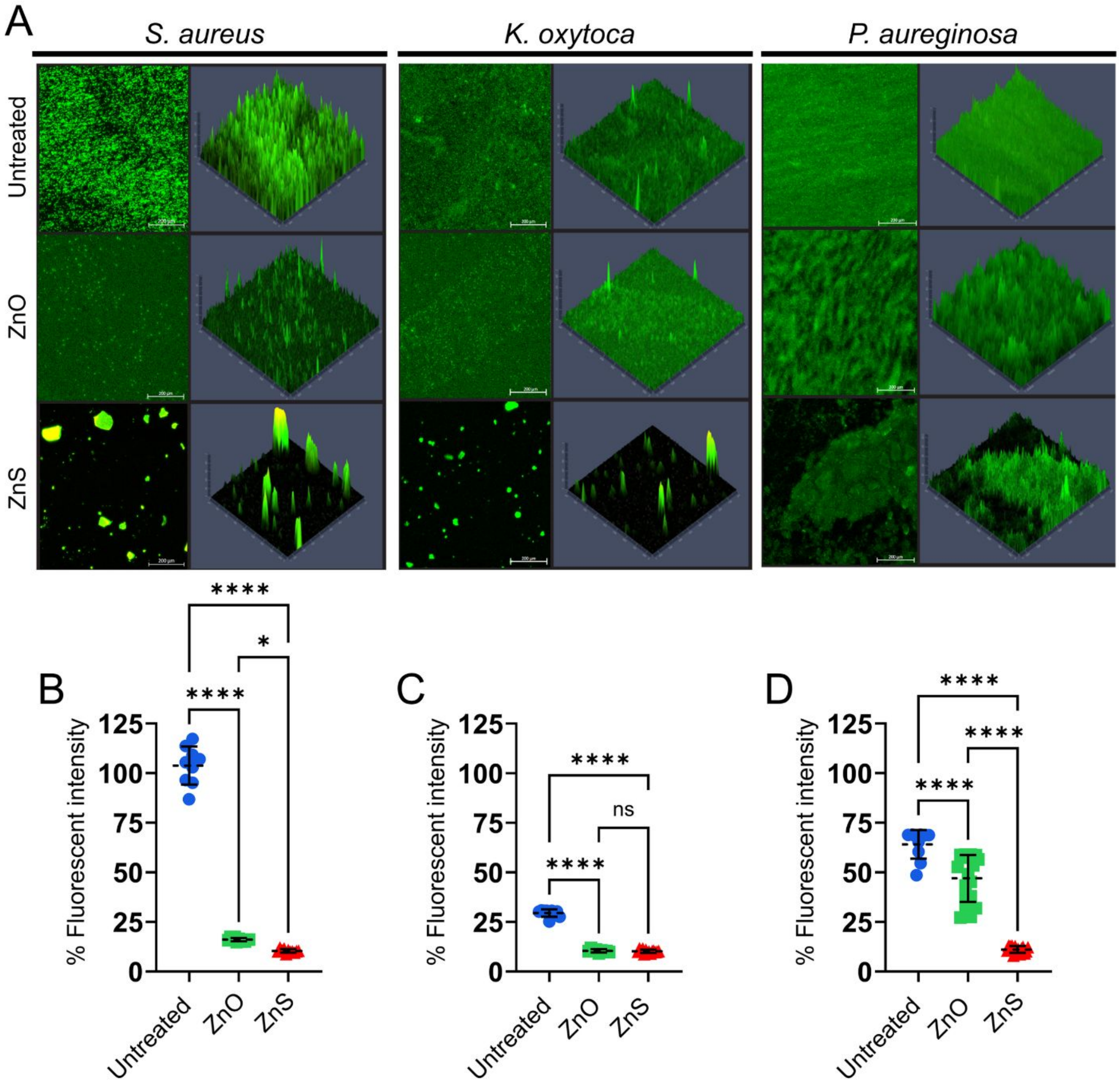

<u>**SFig. 1.**</u> **Zn-NPs inhibit bacterial biofilm formation. (A)** Bacterial biofilms were grown alone (untreated) or with ZnO- or ZnS [0.5 mg/mL (*S. aureus*) or 2 mg/mL (*K. oxytoca* or *P. aeruginosa*)] for 24 h at 37°C. Representative confocal images of untreated (upper panels)-, ZnO-treated (middle panels), or ZnS-treated (lower panels) biofilms [green (SYTO9)] are shown. The thickness and morphology of the bacterial biofilms can be observed in the *Z*-stack reconstruction. The photographs were taken at a ×63 magnification. Scale bars: 200 µm. The percentage (%) of fluorescent intensity for **(B)** *S. aureus*, **(C)** *K. oxytoca*, and **(D)** *P. aeruginosa* biofilm images of bacteria grown in absence or presence of Zn-NPs was quantified using ImageJ software within a defined 100 µm x 100 µm region of interest (ROI). For **B-D**, dashed lines and error bars denote the means and SDs, respectively. Each symbol represents an individual ROI ($n = 30$ for *S. aureus*; $n = 28$ for *K. oxytoca*; $n = 30$ for *P. aeruginosa*). Asterisks denote *P*-value significance ($^*P < 0.05$

and ****$P < 0.0001$) calculated using analysis of variance (ANOVA) and adjusted by use of the Tukey's post-hoc analysis. ns denotes comparisons which are not statistically significant.

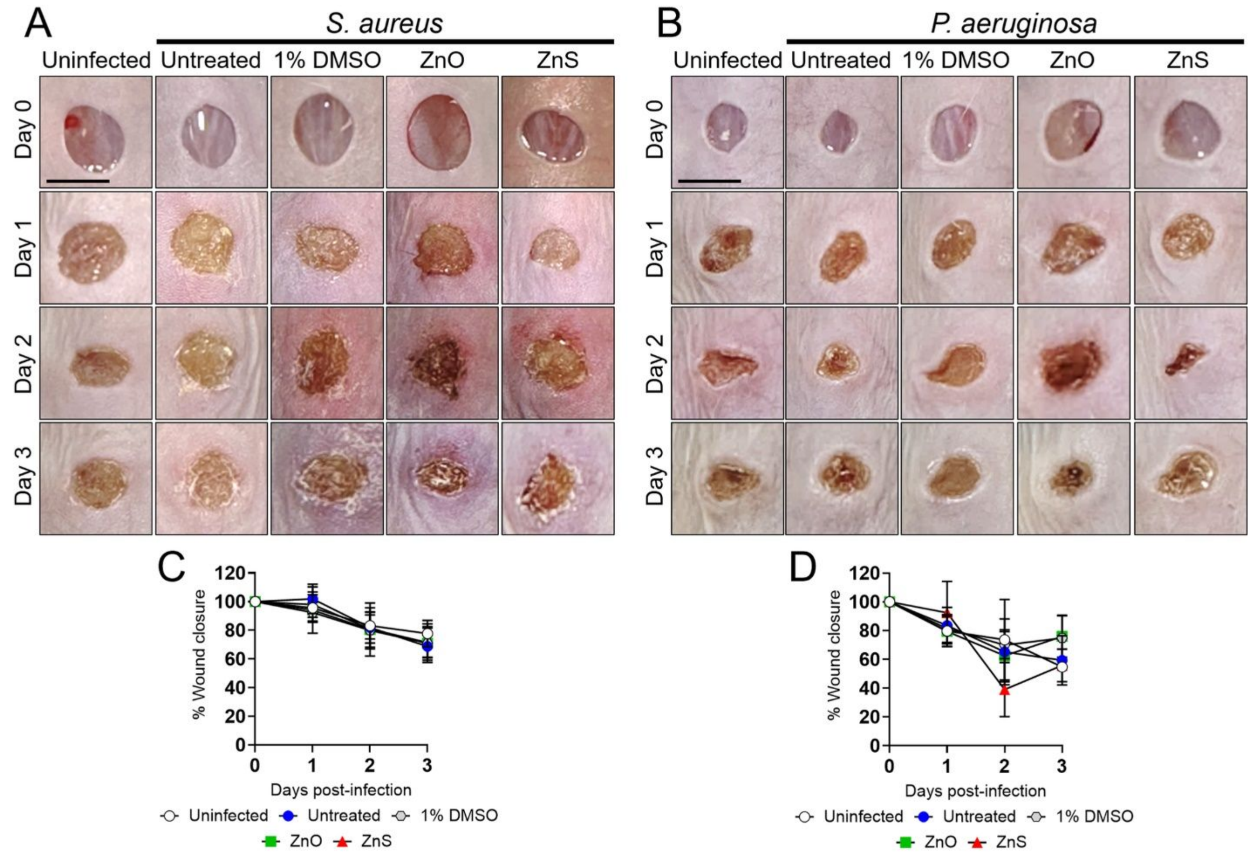

<u>SFig. 2.</u> Zn-NPs do not promote cutaneous wound healing. The wounds of uninfected and **(A)** *S. aureus* 553838 or **(B)** *P. aeruginosa* MRSN 5519 infected Balb/c mice [$n = 4$ per group, a single wound per mouse (untreated or treated twice daily with 1% dimethylsulfoxide (DMSO; NP vehicle), or 5 mg/mL of ZnO- or ZnS-treated for 3-days post-wounding (dpw)] are shown. Mice were infected with 10^7 bacteria. Scale bar: 5 mm. The percentage (%) of wound closure for mice infected with **(C)** *S. aureus* and **(D)** *P. aeruginosa* and treated with Zn-NPs is shown. Each wound was blindly measured with a caliper by 2 independent investigators. Each time point represents the means ($n = 4$ mice per group; a single wound per mouse), and error bars denote SDs. Significance ($P < 0.05$) was calculated by multiple student's t-test analyses.
